# Early-adolescent antibiotic exposure results in mitochondrial and behavioral deficits in adult male mice

**DOI:** 10.1038/s41598-021-92203-1

**Published:** 2021-06-18

**Authors:** Anouk C. Tengeler, Tim L. Emmerzaal, Bram Geenen, Vivienne Verweij, Miranda van Bodegom, Eva Morava, Amanda J. Kiliaan, Tamas Kozicz

**Affiliations:** 1 Department of Medical Imaging, Anatomy, Radboud University Medical Center, Donders Institute for Brain, Cognition & Behaviour, Centre for Medical Neuroscience, Preclinical Imaging Centre PRIME, Nijmegen, The Netherlands; 2 Department of Clinical Genomics, Mayo Clinic, 200 First St. SW, Rochester, MN USA; 3 Department of Biochemistry and Molecular Biology, Mayo Clinic, Rochester, MN USA

**Keywords:** Antimicrobials, Behavioural methods, Metabolomics

## Abstract

Exposure to antibiotic treatment has been associated with increased vulnerability to various psychiatric disorders. However, a research gap exists in understanding how adolescent antibiotic therapy affects behavior and cognition. Many antibiotics that target bacterial translation may also affect mitochondrial translation resulting in impaired mitochondrial function. The brain is one of the most metabolically active organs, and hence is the most vulnerable to impaired mitochondrial function. We hypothesized that exposure to antibiotics during early adolescence would directly affect brain mitochondrial function, and result in altered behavior and cognition. We administered amoxicillin, chloramphenicol, or gentamicin in the drinking water to young adolescent male wild-type mice. Next, we assayed mitochondrial oxidative phosphorylation complex activities in the cerebral cortex, performed behavioral screening and targeted mass spectrometry-based acylcarnitine profiling in the cerebral cortex. We found that mice exposed to chloramphenicol showed increased repetitive and compulsive-like behavior in the marble burying test, an accurate and sensitive assay of anxiety, concomitant with decreased mitochondrial complex IV activity. Our results suggest that only adolescent chloramphenicol exposure leads to impaired brain mitochondrial complex IV function, and could therefore be a candidate driver event for increased anxiety-like and repetitive, compulsive-like behaviors.

## Introduction

The brain is one of the organs that is most affected by impaired mitochondrial function, because it is one of the most metabolically active organs in the body. The brain consumes about 20% of the body’s total available energy while representing only 2% of the total body weight^1–3^. The brain is, therefore, highly sensitive to impaired energy production^4^. The majority of energy used by the brain is generated by mitochondria in the form of Adenosine triphosphate (ATP). Mitochondria are also involved in brain function through the induction of apoptosis and the production of reactive oxygen species (ROS)^4^. Impaired mitochondrial function can, therefore, affect neurodevelopment and brain function due to the central roles of mitochondria in neurogenesis, synaptic plasticity, and neuronal activity^1,5–8^.

Mitochondria share many molecular and cellular characteristics with prokaryotes as a result of their common ancestry. Consequently, many antibiotics that target bacterial translation may also affect mitochondrial translation^9–12^. Even at recommended treatment doses and durations, some antibiotics can damage the mitochondrial DNA, impair quality control, and disrupt energy production^9,13^. For example, chloramphenicol and aminoglycosides like gentamicin, bind to the prokaryotic ribosomal subunits, inhibiting protein synthesis^14,15^. Gentamicin binds to the small 30S prokaryotic ribosomal subunit, while chloramphenicol selectively binds to the large 50S ribosomal subunit^14,15^.

Due to the close ribosomal similarities between prokaryotes and mitochondria, mitochondrial ribosome activity might also be affected by these antibiotics, possibly leading to inhibition of mitochondrial biogenesis^9,16–18^. Dysfunctional mitochondria are reported to be involved in psychiatric disorders and symptoms such as anxiety and depression^4,19–21^. In addition, patients with mitochondrial DNA mutations or mitochondrial diseases often show symptoms of mood disorders^21^. Several classes of antibiotics that affect mitochondrial function, including aminoglycosides and fluoroquinolones, can cause neuropsychiatric adverse effects, such as mania, acute anxiety and hallucinations, in humans^22–24^. A nation-wide population-based Danish study by Köhler-Forsberg et al., has reported increased risks for a wide range of mental disorders, including developmental, behavioral and anxiety disorders, after infections and subsequent antibiotic exposure in childhood and adolescence^25^. Many of the individuals in the cohort of the study by Köhler-Forsberg et al., had severe infections, including, central nervous system (CNS) infections, urological infections, or sepsis^25^. These infections often require long-term treatment with antibiotics^26,27^.

Given the high occurrence of long-term antibiotic exposure, for example in the treatment of meningitis, osteomyelitis, and chronic urinary infections^27–29^, knowledge on the brain pathology following antibiotic drug exposure is of great public health relevance, however, whether such associations reflect effects of the antibiotic drug exposure remains elusive. Therefore, in this study we aimed to investigate the impact of adolescent exposure to antibiotics, like aminoglycosides and chloramphenicol, on cerebral mitochondria and behavior. Chloramphenicol is used to treat severe infections such as bacterial meningitis, osteomyelitis and typhoid fever^30,31^. We hypothesized that certain antibiotics could affect brain mitochondrial function possibly resulting in altered behavior and cognition. To test this hypothesis, we treated young adolescent male wild-type mice with clinically relevant doses of chloramphenicol or gentamicin in their drinking water. Because the effects could also represent general effects of the antibiotic exposure, we also treated one group of mice with amoxicillin^32^. Amoxicillin is part of the beta-lactam family of antibiotics and targets the bacterial cell wall synthesis^33^. Because mitochondria do not possess a cell wall, amoxicillin would not interfere with mitochondrial translation. Next, we assayed mitochondrial oxidative phosphorylation complex activities in the cerebral cortex, performed behavioral screening and targeted mass spectrometry-based acylcarnitine profiling in the brain cortex. We found that adolescent chloramphenicol, but not gentamicin or amoxicillin exposure, without concomitant infection(s), led to impaired brain mitochondrial complex IV function, signs of slower fatty acid metabolism, accompanied by increased anxiety-like and repetitive, compulsive-like behaviors.

## Materials and methods

### Mice

All animal experiments were carried out in accordance to international European ethical standards (European Directive 2010/63/EU) and they were approved by the Veterinary Authority of the Radboud university medical center (Radboudumc; Permit number: RU-DEC 2015-0077) containing a statistical power analysis to minimize group sizes. All applicable (inter)national and institutional guidelines for the care and use of animals were followed and reported in accordance with the ARRIVE guidelines^34^.

**Figure 1:**
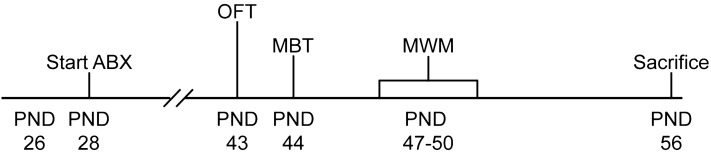
Timeline. Body weight and water consumption were monitored regularly, starting from postnatal day (PND) 26. From PND 28, mice received either amoxicillin, chloramphenicol, gentamicin, or no antibiotics (control group) in drinking water (start ABX). The open field test (OFT) and marble burying test (MBT) were performed on PND 43 and 44, respectively. The Morris water maze (MWM) consisted of 4 days and was assessed on PND 47–50. Mice were sacrificed via cervical dislocation on PND 56.

In total, 40 male, 28 days old C57BL/6J mice were used for this randomized and blind controlled study. The timeline of the study is illustrated in Fig. 1. Mice were group-housed with two mice in digital ventilated cages (DVC; Tecniplast S.P.A., Buguggiate (VA), Italy) and housed under standard laboratory conditions. Animals were randomly assigned to one of the four experimental groups (Control, Amoxicillin, Chloramphenicol, and Gentamicin; N = 10 per group). Body weight of all mice was measured weekly.

### Antibiotic exposure

Mice were administered the following antibiotics dissolved in their drinking water as indicated: amoxicillin (0.5 g/l; A8523), chloramphenicol (0.5 g/l; C0378), or gentamicin (0.035 g/l; G1914) for 4 weeks. All antibiotics were purchased from Sigma–Aldrich, St. Louis, MO. The doses in the drinking water correspond to the approximately daily doses given to children normalized based on body weight (20–25 g) and expected water consumption (5 ml per day)^35^. One group of mice did not receive antibiotics and served as control group. Drinking water was not sweetened to mask the taste of the antibiotics because sugar also affects body weight, behavior, and cognition. Instead, water consumption was monitored for each pair of mice by measuring water volume three times per week to prevent dehydration, and to ensure that all animals drank equal amounts of water. Drinking water of all experimental groups was refreshed three times per week.

### Behavioral tests

Open Field Test (OFT) was used to evaluate locomotion and explorative behavior. Mice were placed in the center of the square open field (45 × 45 × 25 cm) with transparent Plexiglas walls, and allowed to freely explore the area for 10 min. The trials were videotaped using a camera that was positioned above the open field area. Exploration activity in the center (20 × 20 cm) and corners (10 × 10 cm) was automatically scored and quantified using Ethovision XT10.1 (Noldus, Wageningen, The Netherlands).

We used the marble burying test (MBT) as an accurate and sensitive assay of anxiety-like, repetitive and compulsive-like behaviors in rodents^36^. Mice were placed in a clean conventional-sized cage preloaded with 3 cm clean bedding and 15 evenly spaced black glass marbles with a 14 mm diameter. Mice were allowed to explore the cage for 30 min, and thereafter the cage was photographed. Three evaluators counted the number of marbles that were covered by bedding at least two-thirds of their size between trials, marbles and cages were cleaned with 70% Ethanol.

The Morris water maze (MWM) was used to assess spatial learning and memory. During the acquisition phase, the mice were trained to find the platform in a circular pool (diameter 108 cm) that was filled with water (21–22 °C), made opaque by adding milk powder. The platform (diameter 8 cm) was submerged 1 cm below the water surface and located in the north-east quadrant of the pool. Mice performed 4 acquisition trials per day starting from four different cardinal points (South, North, East, West; maximal swimming time 120 s; 30 s on platform; inter-trial interval 60 min) during 4 consecutive days. Visual cues were present on the four walls surrounding the pool at a distance of 0.5 m. All trials were recorded and latency to find the platform (s) was used as a measure for spatial learning. Mice performed a single probe trial at the end of day 4 of acquisition, in which the platform was removed from the pool. During this probe trial, the mice were allowed to swim freely for 120 s. Trials were recorded and analyzed with EthoVision XT 10.1 (Noldus, Wageningen, the Netherlands).

### Mitochondrial analysis

Mice were sacrificed via cervical dislocation without anesthesia after which the brains were rapidly removed and dissected. The whole brain cortex was snap frozen in liquid nitrogen. A crude homogenate of the cortex was made with a glass-glass potter tube in SEF buffer (0.25 M sucrose, 2 mM K-EDTA, 10 mM phosphate buffer, pH 7.4) to obtain a 5% w/v homogenate. The homogenized samples were centrifuged at 600×*g* for 10 min at 2 °C. The supernatant was aliquoted, snap frozen in liquid nitrogen, and stored at − 80 °C for mitochondrial complex activity measurements.

Protein levels and enzyme activities of the individual complexes of the respiratory chain (complex I to IV), succinate: Cytochrome C oxidoreductase (SCC), and citrate synthase (CS; a marker of the number of mitochondria^37^) in the 600 g supernatant were measured spectrophotometrically, after three freeze–thaw cycles on a KoneLab 20XT analyzer (Thermo Scientific) following standard procedures^38^. These enzyme assays are based on previously described methods^37,39–42^. All measurements were normalized to both CS activity and protein levels.

### Analysis of acylcarnitine metabolites

The whole brain cortex was also used to measure several different acylcarnitine metabolites using an UPLC-MS method as previously described^43^. In short, 5 mg of pulverized tissue was homogenized in 50 µl of PBS before 25 µl of deuterated labeled internal standards was added. Proteins were removed by adding a solution of methanol/dichloromethane (v/v, 600 µl) to the sample mixture. The sample was centrifuged at 18,000×*g* for 15 min at 4 °C, and then the supernatant was transferred to a 1 dram vial, and dried under N2 stream. Samples were reconstituted and analyzed on a Waters Acquity UPLC system (Milford, MA) coupled with a Thermo Quantiva tandem mass spectrometer (West Palm Beach, FL) in positive (H)ESI mode. Concentrations of carnitine (162.1 > 85.0 m/z), acetylcarnitine (204.1 > 85.0 m/z), propionylcarnitine (218.1 > 85.0 m/z), butyrylcarnitine (232.1 > 85.0 m/z), isovalerylcarnitine (246.1 > 85.0 m/z), octanoylcarnitine (288.2 > 85.0 m/z), lauroylcarnitine (344.3 > 85.0 m/z), myristoylcarnitine 372.3 > 85.0 m/z), palmitoylcarnitine (400.4 > 85.0 m/z), oleoylcarnitine (426.4 > 85.0 m/z), and stearoylcarnitine (438.4 > 85.0 m/z) were measured against a 11-point calibration curve that underwent the same preparation.

### Statistical analysis

Data were analyzed using IBM SPSS for Windows 25.0 software (SPSS Inc., Chicago, IL, USA). The latency to find the platform during the acquisition phase of the MWM, body weight, and water consumption were analyzed using repeated measures ANOVA. A MANOVA was performed for the parameters of the open field test, probe phase of the MWM, acylcarnitine, and mitochondrial complex activity measurements as dependent variables. ANOVA’s were followed by Dunnett’s multiple comparison test to compare the control group (mice that received water) with the treatment groups. A Kruskall-Wallis with Dunn’s pairwise tests, and Bonferroni post hoc correction was used to analyze the marbles buried in the marble burying test. Statistical outliers were removed from the dataset. Significant MANOVAs and the acylcarnitine results were followed up by a discriminant function analysis (DFA). This DFA was performed with a stepwise approach and Mahalanobis distance as method.

## Results

### Administration of antibiotics in drinking water had no effects on water consumption or body weight

**Figure 2:**
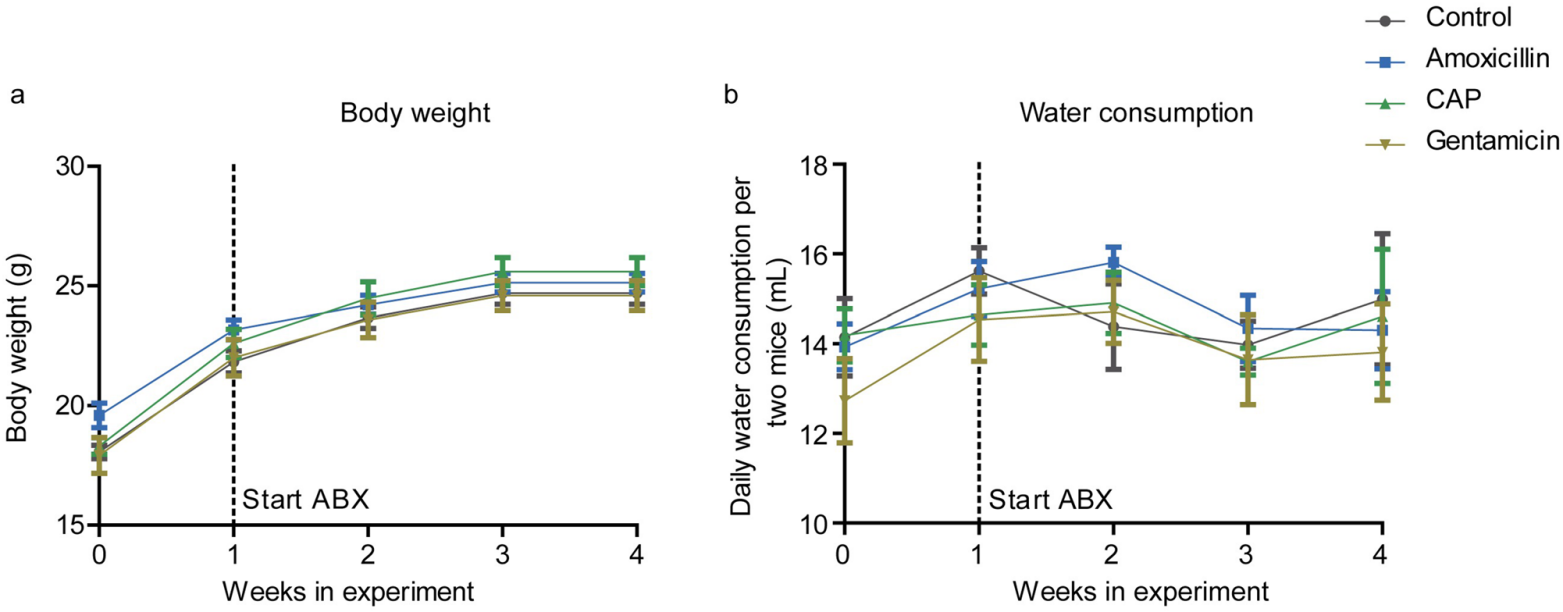
Body weight and water consumption. The body weight of all mice was measured weekly before start of antibiotics administration (week 0) and after (week 1–4). Mice gained weight over the weeks (p < 0.001). No differences between the experimental groups were observed (**A**). Daily water consumption per two mice before treatment with antibiotics (week 0) and after (week 1–4). No differences between the groups were detected (**B**). CAP, chloramphenicol. N = 10 per group. Data are presented as mean ± SEM.

Body weight and water consumption were measured to evaluate the effects of antibiotics on these parameters. Initial body weight did not differ between the experimental groups, and all mice gained body weight over the next 4 weeks [F(4,144) = 431.65, p < 0.001; Fig. 2a]. No differences in body weight gain between the experimental groups were detected.

The pairs of mice consumed 13.8 ± 0.3 mL water daily before start of the antibiotics administration. No differences in water consumption from week 0 to week 4 were observed. Water consumption was comparable between the experimental groups (Fig. 2b).

### Chloramphenicol treatment affects repetitive and anxiety-related behavior in mice

**Figure 3:**
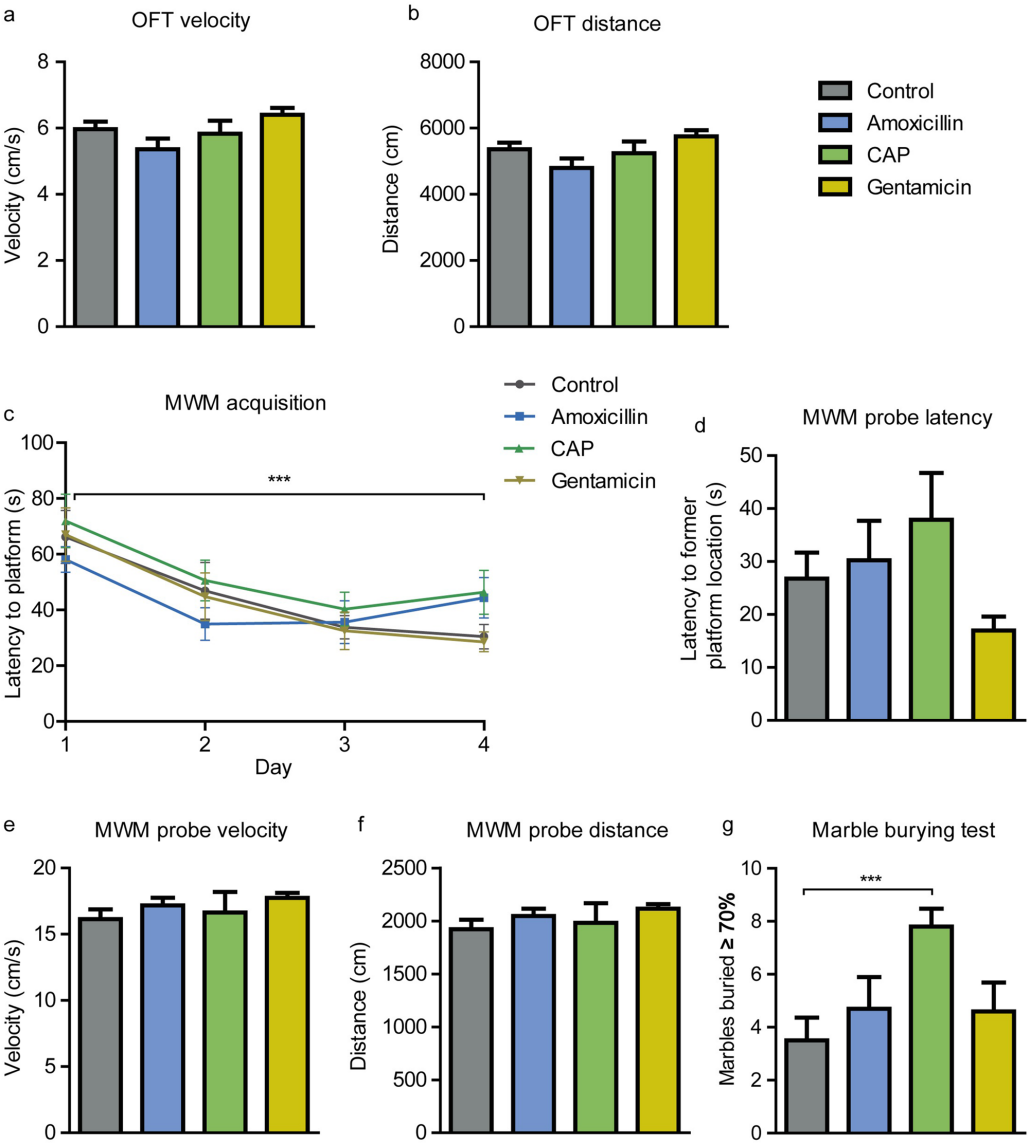
Behavior and cognition assessed with the OFT, MWM and MBT. Mean walking velocity (**A**) and total distance walked (**B**) during the OFT. No differences between the experimental groups were detected. Spatial learning assessed with the MWM in a 4-day acquisition phase. All groups showed a learning curve, but no differences between the groups were observed (**C**). The latency to go to the former platform location in the probe phase of the MWM (**D**), and the mean swimming velocity (**E**) and total swimming distance (**F**) were not affected by the antibiotics assessed. Buried marbles (i.e., more than two-thirds of its surface area) were scored by 3 task scorers (**G**). The scores for each mouse were averaged for all scorers. Mice that were administered chloramphenicol buried more marbles than control mice (p = 0.019). CAP, chloramphenicol. N = 10 per group. Data are presented as mean ± SEM.

We examined locomotion, explorative behavior, spatial memory and spatial learning in the OFT and MWM. We found no differences in locomotion and explorative behavior in the OFT (Fig. 3a,b). Spatial learning and memory of the mice were tested with the MWM test. All mice learned to find the hidden platform during the acquisition phase, and this was not affected by any of the treatments (Fig. 3c). During the probe phase of the MWM, the platform was removed. No differences between the groups were observed in the latency to go to the former platform location (Fig. 3d), even after controlling for mean swimming velocity. No differences in mean swimming velocity (Fig. 3e) and the total swimming distance (Fig. 3f) were observed as well. These data suggest that treating rodents with either amoxicillin, chloramphenicol or gentamicin does not influence overall activity, exploration and spatial learning and memory.

An accurate and sensitive assay of anxiety-like, repetitive and compulsive-like behaviors in mice was performed by using the marble burying test^36^. Marble burying behavior was significantly affected by antibiotic administration (H(3) = 9.76, p < 0.05; Fig. 3g). Dunn’s pairwise tests revealed a significant increase in marbles buried by mice treated with chloramphenicol compared to the control group (p = 0.019). Gentamicin and amoxicillin did not affect marble burying behavior.

### Chloramphenicol treatment results in decreased activity of the respiratory chain complex IV

**Figure 4:**
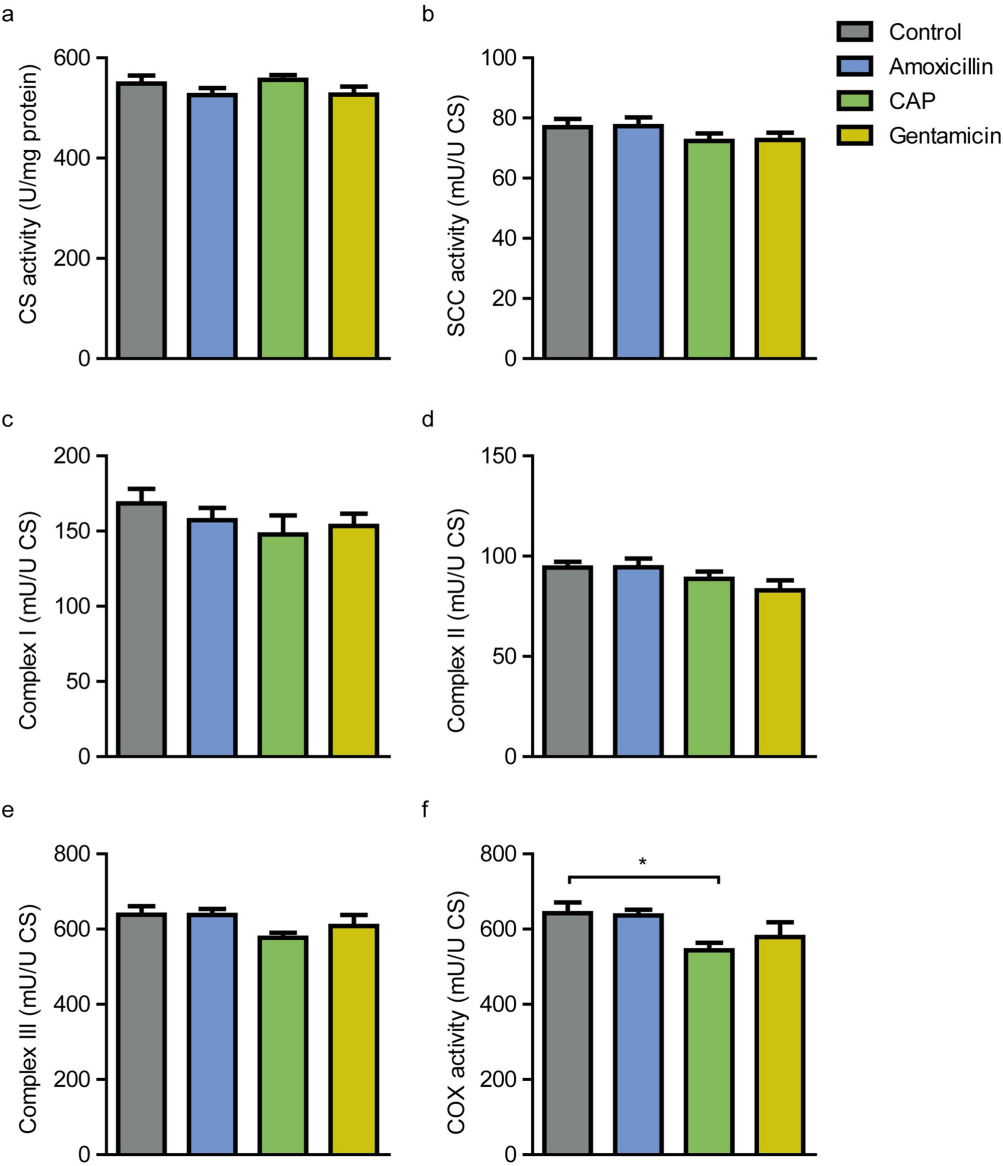
Activity of mitochondrial complexes. CS activity was used to normalize all measurements. CS activity did not differ between the groups (**A**). SCC activity (**B**), Complex I activity (**C**), complex II activity (**D**), complex III activity (**E**), and COX activity (**F**) were measured in all mice. COX activity, used to measure complex IV activity, was decreased in mice that were administered chloramphenicol (p = 0.035). CAP, chloramphenicol; CS, citrate synthase; COX, cytochrome c oxidase. N = 9 (control) and N = 10 (all other groups). Data are presented as mean ± SEM.

Mitochondrial complex activity was measured in the cortex of all mice. One control mouse was considered a statistical outlier and was removed from further analyses. All measurements were normalized against CS activity per mg protein which did not differ between the experimental groups (Fig. 4a). The activity of respiratory chain complexes I, II, and III, and SCC activity were not affected by the studied antibiotics (Fig. 4b–e). However, cytochrome C oxidase (COX), which was used to measure complex IV activity, showed a reduced activity in mice treated with chloramphenicol compared to vehicle treated mice (p = 0.035; Fig. 4f). These results reveal that chloramphenicol, besides its effect on behavior, reduces brain mitochondrial complex IV function.

The MANOVA was followed up with a discriminant function analysis (DFA) to assess whether groups differ along a linear combination of outcome variables. Predictors were activity of mitochondrial complex IV, and number of marbles buried in the MBT. The DFA revealed that activity of mitochondrial complex IV was the main predictor that explained 100% of variance, canonical R^2^ = 0.457. This discriminant function significantly differentiated the antibiotics groups, Λ = 0.791, χ^2^(3) = 8.323, p = 0.04.

The classification results showed that 25.6% of all cases were correctly classified; 40% of mice treated with chloramphenicol were classified correctly, whereas 11.1% of control mice were correctly predicted. Of amoxicillin-treated and gentamicin-treated mice, 0% and 20% were correctly classified, respectively.

### Antibiotic treatment alters carnitine metabolism

**Figure 5:**
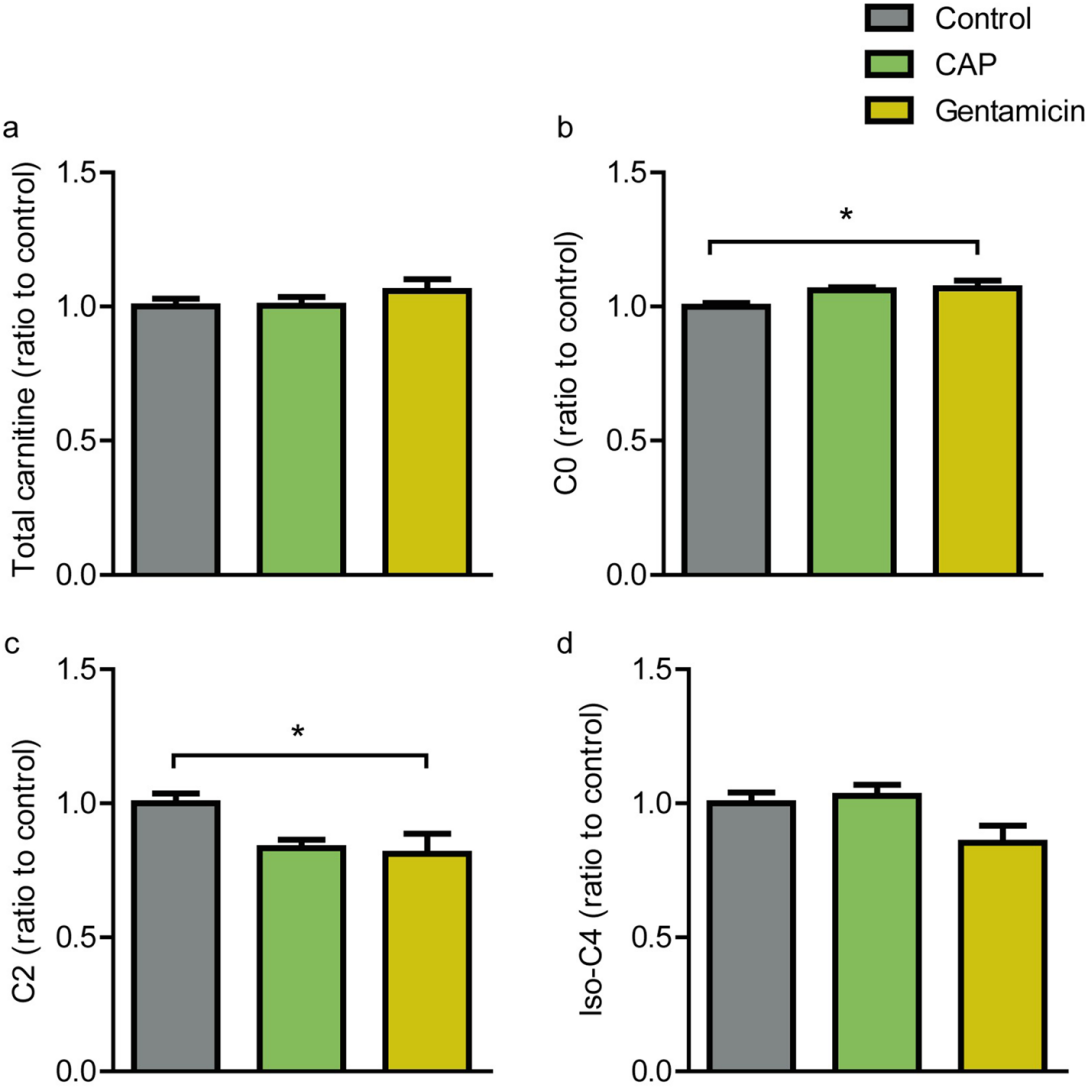
Acylcarnitine metabolite profile. Total carnitine concentration (**A**) was used to normalize all individual acylcarnitine measurements. Ratio of free carnitine (**B**), Acetylcarnitine (**C**), and isobutyrylcarnitine (**D**). CAP, chloramphenicol. N = 10 mice per group. Data are presented as mean ± SEM.

Acylcarnitine metabolism is a classical marker of mitochondrial dysfunction. Therefore, next we explored whether acylcarnitine metabolism is altered in chloramphenicol exposed mice with decreased COX function. We assessed free carnitine (C0) and 11 acylcarnitine species in control, chloramphenicol, and gentamicin treated mice. All results were corrected to total carnitine concentrations to normalize the data (Fig. 5a). We found significant differences between treatment groups in free carnitine (C0; p = 0.033), acetylcarnitine (C2; p = 0.031), and isobutyrylcarnitine (Iso-C4; p = 0.042). Post hoc multiple comparisons revealed that gentamicin treatment increased C0 (p = 0.032; Fig. 5b) concentrations, while chloramphenicol treatment only tended to increase C0 abundances (p = 0.059). Conversely, post hoc comparisons revealed that gentamicin treatment negatively affected C2 concentrations (p = 0.030; Fig. 5c), while chloramphenicol only tended to decrease C2 abundance (p = 0.056). Although Iso-C4 concentrations varied significantly between the different treatments, this was not influenced by antibiotic treatment compared to the control group (Fig. 5d). These results identify brain acylcarnitine metabolism derangements in mice treated with gentamicin.

**Figure 6:**
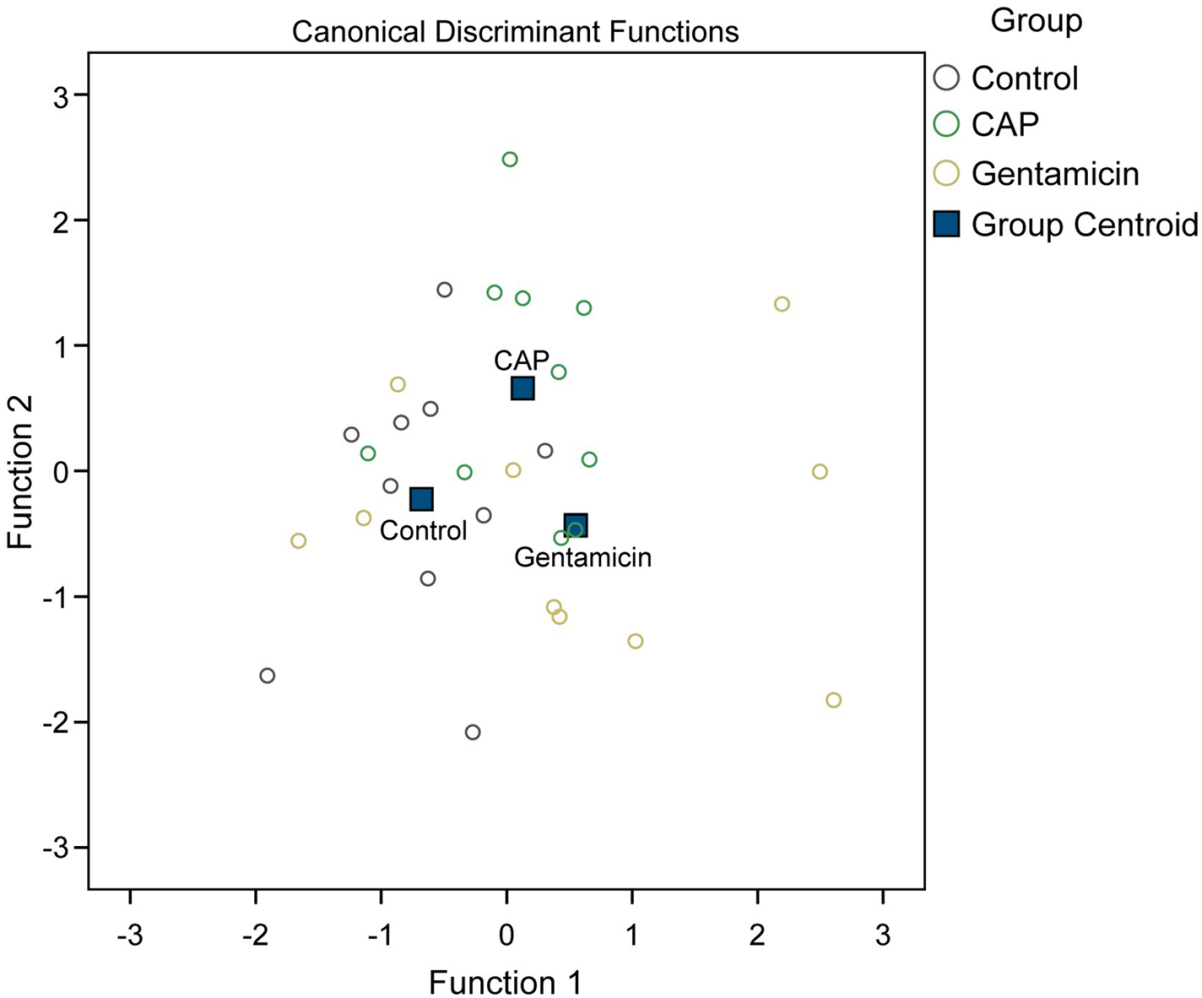
Discriminant function analysis. Canonical variables plot of the discriminant function analysis that classifies control (blue circles), chloramphenicol (green circles) and gentamicin (yellow circles) samples based on the acylcarnitine profile. N = 10 per group.

These results were followed up by a stepwise DFA that showed that both Iso-C4 and C0 were the main predictors for differentiation between the groups. The two discriminant functions explained 53.7% (canonical R^2^ = 0.474) and 46.3% (canonical R^2^ = 0.447) of the variance, respectively. Combining these discriminant functions show that they significantly differentiate the groups, Λ = 0.621, χ^2^ (4) = 12.625, p = 0.013 (Fig. 6).

## Discussion

Despite the high occurrence of exposure to antibiotics during adolescence and its association with increased risk for developing psychopathology^25,44,45^, an important research gap exists in understanding how antibiotic therapy during adolescence could lead to increased vulnerability to developing psychopathology. Finding potential driver events explaining this association remains of public health relevance.

Here we show that adolescent exposure to chloramphenicol but not to amoxicillin or gentamicin is associated with decreased mitochondrial complex IV activity in the brain, and with increased anxiety-like, repetitive and compulsive-like behaviors in male mice. Stepwise DFA confirmed that mice exposed to chloramphenicol could clearly be discriminated from the other experimental groups using one predictor: activity of mitochondrial complex IV. Of mice that were treated with chloramphenicol, 70% could be classified correctly.

Complex IV is a highly regulated enzyme that is involved in the reduction of O_2_ to H_2_O^46^. As the final electron carrier in the electron transport chain, complex IV acts as the rate-limiting step for electron transfer. Complex IV activity is therefore an indication of the oxidative capacity of the cells^47^. Mitochondrial complex I, II and III were not affected in this study. This has also been reported in a study in which the effects of chloramphenicol on bovine aortic endothelial cells were explored. In that study, chloramphenicol also inhibited mitochondrial protein synthesis resulting in a decrease of complex IV activity^48^. Besides that, they also found no effects of chloramphenicol on complex II or complex III. Another in vitro study showed that chloramphenicol lowered the expression of the mitochondrial DNA (mtDNA)-encoded COX 1 subunit of complex IV, resulting in a reduction of COX1 protein levels^49^. Chloramphenicol, and other antibiotics that target ribosome function, inhibits translation of protein encoded by the mitochondrial, but not the nuclear, genome^32^. A relative imbalance between the expression of nuclear and mitochondrially encoded proteins of the subunits of the mitochondrial complexes, termed mitonuclear protein imbalance, could drive mitochondrial complex instability^50,51^, possible resulting in decreased complex IV activity. However, the exact mechanisms through which chloramphenicol impacts complex IV activity in vivo remains to be explored in future studies.

We did not detect an effect of gentamicin on mitochondrial function in the brain cortex of the mice. It is possible that this is due to differences in permeability across the blood–brain barrier. Whereas chloramphenicol readily crosses the blood–brain barrier, the permeability of this barrier to gentamicin and other aminoglycosides is shown to be poor^52^. Finally, administration of amoxicillin did not affect brain mitochondrial functions either. This is consistent with its mode of action in bacteria since it disrupts the bacterial cell wall and therefore would not affect mitochondrial function.

The brain has the highest mitochondrial energy demand of any organ. Multiple pieces of preclinical and clinical evidence suggest that brain mitochondrial function is impaired in the pathobiology of neuropsychiatric presentations^1,53–56^. Our results therefore suggest that adolescent chloramphenicol exposure alone, without infection(s) leads to impaired brain mitochondrial complex IV function that could be a candidate driver event for increased anxiety, repetitive and compulsive-like behaviors, although the causality of this relationship should be investigated in future studies.

Carnitine, that conjugates with acyl-coenzyme A (acyl-CoA) to form acylcarnitine, is vital for transport of fatty acids into mitochondria for subsequent β-oxidation resulting in acetyl-CoA and electron carriers that deliver electrons to the electron transport chain^57,58^. Altered plasma concentrations of acylcarnitines are suggested markers of impaired metabolism, although acylcarnitine profiles are usually nonspecific^59^. Acetyl-CoA can be converted to acetylcarnitine, which is the shortest acylcarnitine. Acetylcarnitine can cross the mitochondrial membrane and enables mitochondrial efflux of excess acyl groups. We found that acetylcamitine (C2) was decreased during gentamicin treatment. This was accompanied by an increase in free-carnitine levels as a possible consequence of decrease in acyl group transfer and metabolism.

Chloramphenicol, an antibiotic which is able to cross the blood–brain barrier, affected mitochondrial complex IV activity, whereas gentamicin, which cannot easily pass the blood–brain barrier, only impacted the acylcarnitine metabolites profile, but not complex IV activity in the brain. This could suggest that chloramphenicol directly affects cerebral mitochondria, while the effects of gentamicin are indirect, for example via the gut microbiota. It has been extensively discussed in the literature that the use of both single and multiple antibiotic regimens change the gut microbiota and affects the brain-gut axis^60–62^. In this study we did not assess the microbiota, therefore, whether antibiotic exposure directly alters mitochondrial function and the animal’s behavior, or indirectly, for example via altering the gut microbiome, remains to be explored in future studies.

When interpreting our results, one should also consider that chloramphenicol is capable of inducing oxidative stress. Although not measured in this study, high levels of reactive oxygen species (ROS) and nitric oxide (NO), for instance in case of mitochondrial complex IV dysfunction, are also associated with high anxiety and compulsive behavior in mice^63,64^.

This study is not without technical considerations. Serial oral gavage is the most often used method to administer precise doses of antibiotics to mice. This route of administration is also associated with adverse effects like stress^13^ that could be a potential confound in the outcome of behavioral tests. We chose to administer antibiotics via unsweetened drinking water to mice. Although this could be seen as a potential limitation (animals consume less water), our results show that administration of antibiotics via unsweetened drinking water did not alter water consumption. This study only used male mice. Several psychopathologies have been associated with early antibiotics treatment, such as attention deficit hyperactivity disorder (ADHD) and autism spectrum disorder (ASD). These disorders are more prevalent in males than females, although this may be a consequence of diagnostic bias, due to differences in symptom presentation^65,66^. It is, however, of utmost importance to include female mice in future studies to delineate the impact of antibiotic treatment on brain pathology. As dysfunctional mitochondria are reported to be involved in psychiatric disorders like anxiety disorders and depression^4,19–21^, it would be worthwhile to include behavioral tests to explore depression-like behavior as well in further studies. Future studies should also evaluate effects of the antibiotics at a later time point, to differentiate between immediate and long-lasting effects of the antibiotics exposure. Also, in this study we investigated mitochondrial function in the cerebral cortex of the mice. Future research may consider to explore mitochondrial function in other brain regions as well. Additionally, mitochondrial function in other tissues, for instance muscle or liver, might be worthwhile to study as well, because peripheral effects could be partially responsible for the effects of chloramphenicol on behavior.

Exposure to infections and concomitant antibiotic treatment are associated with increased vulnerability to develop various complex neuropsychiatric disorders. However, whether such increased risk for developing neuropsychopathology reflects effects of the antibiotic drug exposure, that of the infections or both, remains incompletely defined. In this study, we focused on the effects of antibiotic treatment in the absence of infections. The link between infections and complex neuropsychiatric disorders, and the potential modulating role of antibiotics, remains to be studied in future research.

In conclusion, we present evidence that adolescent exposure to chloramphenicol leads to bioenergetic scars that may be implicated in the risk for developing psychopathology. The collateral damage of adolescent antibiotic exposure could be long lasting. More knowledge on the impact of antibiotics exposure on mitochondrial function could help us develop strategies to reduce the impact of adolescent antibiotics exposure on the brain.

## Data Availability

The datasets generated during and/or analyzed during the current study are available from the corresponding author on reasonable request.
